# Exploring the potential for improving material utilization efficiency to secure lithium supply for China's battery supply chain

**DOI:** 10.1016/j.fmre.2022.12.008

**Published:** 2022-12-25

**Authors:** Xin Sun, Han Hao, Yong Geng, Zongwei Liu, Fuquan Zhao

**Affiliations:** a State Key Laboratory of Automotive Safety and Energy, Tsinghua University, Beijing 100084, China; b Belfer Center for Science and International Affairs, Harvard University, Cambridge, MA 02138, United States; c Tsinghua-Rio Tinto Joint Research Center for Resources Energy and Sustainable Development, Tsinghua University, Beijing 100084, China; d School of International and Public Affairs, Shanghai Jiao Tong University, Shanghai 200240, China

**Keywords:** Lithium, Lithium-ion battery, Critical material, Supply chain, Material flow analysis

## Abstract

Lithium-ion battery (LIB) is the key technology for climate change mitigation. The sustainability of LIB supply chain has caused widespread concern since the material utilization efficiency of LIB supply chain has not been well investigated. This study aims to fill this research gap by conducting a dynamic material flow analysis of lithium in China from 2015 to 2021. Results indicate that within the temporal boundary, lithium flow and in-use stock grew significantly in China due to the rapid development of the EV market, with lithium flow in domestic production of basic chemicals increasing by 614% to 100 kt, end-use consumption increasing by 160% to 35 kt, and in-use stock increasing by 62% to 195 kt. China has been a net importer of lithium, of which cumulative imports and exports were 343 kt and 169 kt, respectively. In addition, 103 kt of lithium was converted to inventories or was lost during the processing from 2015 to 2021. By optimizing inventory and processing, developing substitutes for lithium for non-battery applications, and improving lithium recycling, China's net import dependency of lithium could be reduced from 27%-86% to 0%-16%. Our study demonstrates that it is urgent to improve material utilization efficiency so that the lithium resource supply can be secured.

## Graphical abstract

**Figure:**
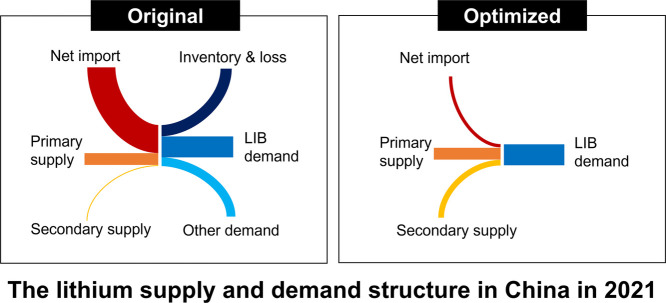

## 1 Introduction

To respond to climate change, many countries and enterprises have set up ambitious plans to achieve zero-carbon energy transition [1], [2], [3], [4], [5]. Among different clean energy technologies, lithium-ion battery (LIB) is one of the most important products as the major power source in consumer electronics and electric vehicles (EVs), promoting energy storage in the renewable power system [6,7]. Global LIB shipments were over 500 GWh in 2021 and are expected to have a 10–20 fold increase by 2050 [8,9]. The rapid expansion of the LIB market amplifies the potential risks in its upstream supply chain, which consists of many “critical materials”, e.g., lithium, cobalt, nickel, and graphite [10], [11], [12]. These materials are deemed to be critical because of their high supply concentration, significant environmental and social impacts, high importance to the performance of final products, low substitutability, and other factors [13], [14], [15], [16].

**Fig. 1:**
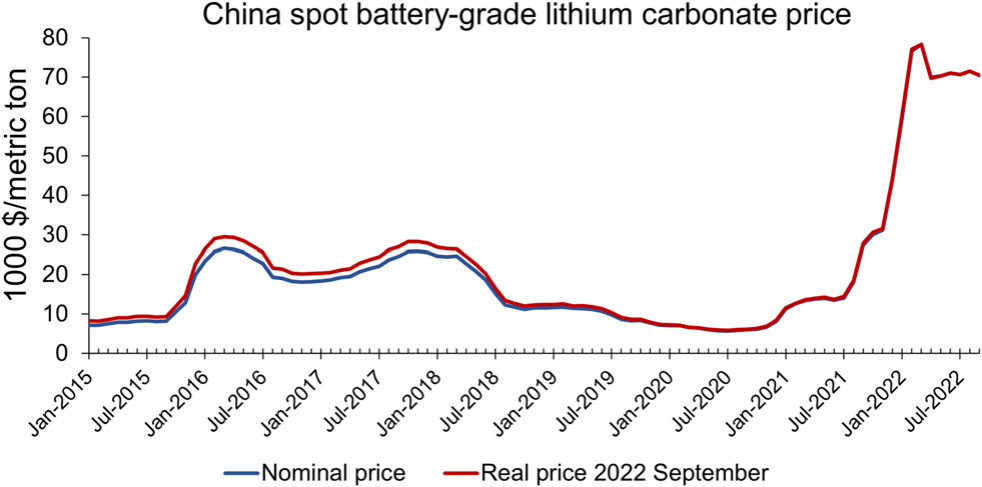
**Monthly lithium price in China**. The real price is obtained by adjusting the nominal price using the monthly consumer price index with 2022 September as the base month. Data from Ref. [22].

With the continuous growth of LIB consumption, the conflicts between unsustainable issues and the stability of battery-related critical material supply are increasingly prominent [9,17]. The over 10-fold increase of lithium price from September 2021 is compelling evidence of this conflict (Fig. 1), leading to global concerns. One significant driver of this price crisis is the distorted raw material demand caused by the overcapacity of downstream commodities [18]. The excess demand posed a huge challenge for lithium suppliers, especially for countries that are highly relying on the external supply of critical materials, e.g., China [19]. Due to a lack of high-quality domestic reserves and large demand for downstream commodities, China has long been dependent on importing such raw materials. Such a trend will continue in the near future [20,21]. Soaring lithium prices have greatly influenced the operations of China's lithium industry chain. It is necessary to establish a highly resilient supply-demand system through improved material utilization efficiency so that such raw material price crises can be avoided. Consequently, there is an urgent need to uncover the characteristics and mechanisms of lithium metabolism along the entire battery industry chain.

Material flow analysis (MFA) is a commonly-used and effective tool to serve this purpose [23]. Many MFA studies have been conducted on battery-related critical metals at specific element levels, including lithium [24], [25], [26], [27], [28], [29], [30], [31], cobalt [32], [33], [34], [35], [36], [37], [38], [39], [40], [41], [42], [43], nickel [44], [45], [46], [47], [48], manganese [49], [50], [51], and graphite [11], or at the battery level [52], [53], [54]. These previous MFA studies have provided valuable information. But research gaps still exist. Because other critical materials besides lithium can be absent in the LIB chemistry system, MFA of these materials could not provide a comprehensive figure of the LIB industry chain. Existing MFA studies of lithium all have a limited temporal boundary which is before the year 2019, which cannot reflect the impacts of skyrocketing EV market growth and the COVID-19 pandemic from 2020 onwards.

Moreover, due to the longstanding data transparency issues in the global lithium industry and poor understanding of the technical properties of lithium-containing commodities, few studies have considered the inventory and losses during the processing stage but assumed that the total consumption of raw materials is equal to the total production of downstream commodities. The status quo and potential for improving material utilization efficiency of the LIB industry chain are still unclear.

Based upon such realities, this study aims to trace lithium flows and stocks, including lithium production, consumption, international trade, inventory changes, disposal, and recycling, in China from 2015 to 2021 by employing the bottom-to-up MFA method. The academic contribution of this study is that we analyzed the issue of inventories and losses in all processes covered in the lithium anthroposphere life cycle. We calculated the lithium contents in various lithium-containing commodities, including minerals, chemicals, LIBs, EVs, etc. Then the lithium flow into the apparent consumption and production was traced separately. Thus, the difference between the apparent consumption of raw materials and the production of downstream commodities, which are the lithium flows into inventories and losses, could be observed. Based on the up-to-date analysis of material utilization efficiency, we identified the future pathways for optimizing China's LIB industry chain.

The remainder of the paper is organized as below. Section 2 details research methods and data for tracing lithium flows and stocks. Section 3 presents research results. Section 4 provides policy recommendations accordingly with further analyses of these results. Section 5 summarizes the conclusions of this study and points out future research directions.

## 2 Methods

### 2.1 System definition

**Fig. 2:**
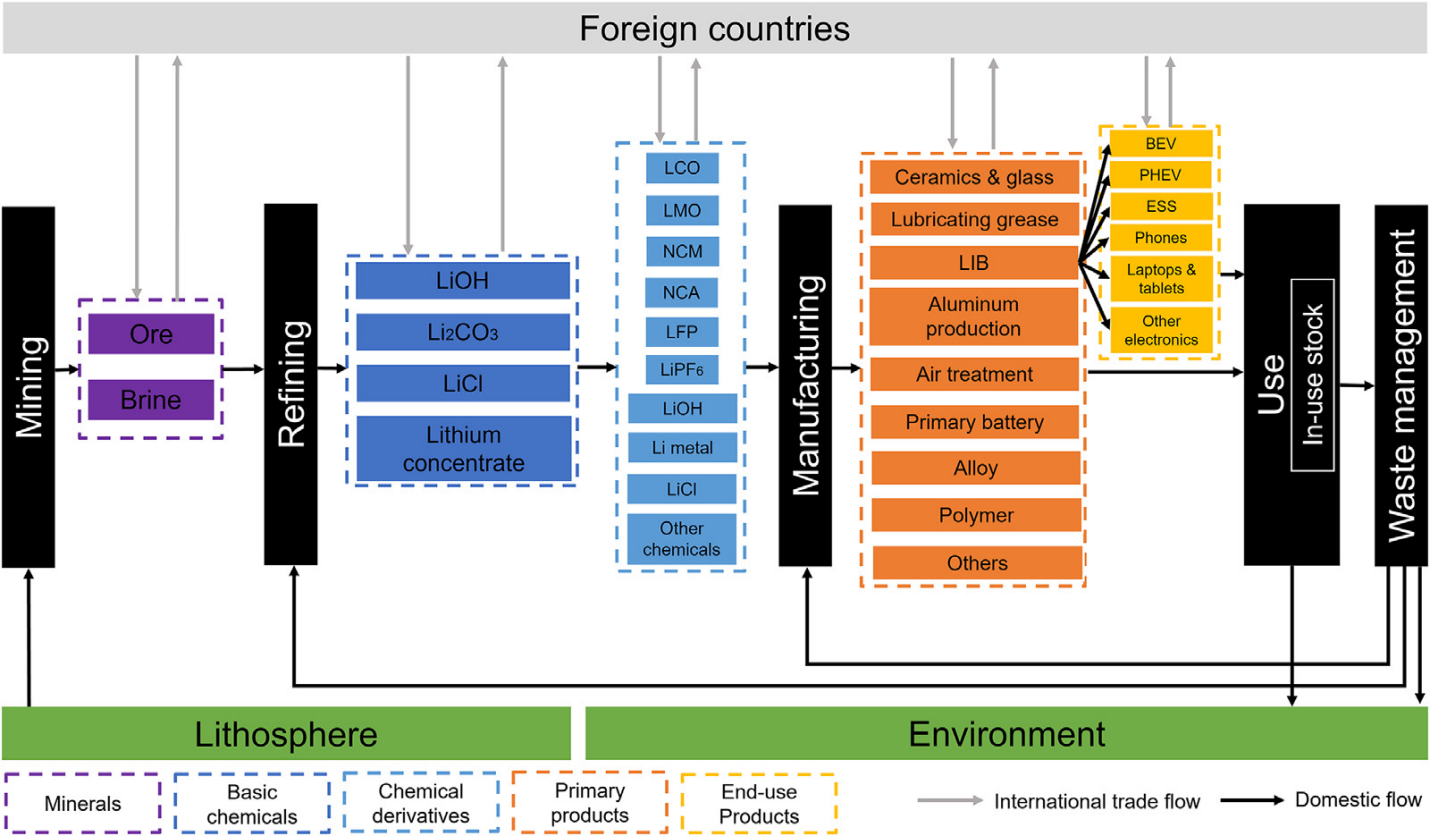
**Conceptual framework of lithium anthropogenic cycle**. The black rectangles represent various stages. The colored rectangles represent involved commodities. Arrows with gray and black colors represent international trade flows and domestic flows, respectively. Li_2_CO_3_, lithium carbonate. LiOH, lithium hydroxide. LiCl, lithium chloride. NCM, lithium nickel cobalt manganese oxide. NCA, lithium nickel cobalt aluminum oxide. LCO, lithium cobalt oxide. LMO, lithium manganese oxide. LFP, lithium iron phosphate. LiPF_6_, lithium hexafluorophosphate. Li metal, lithium metal. LIB, lithium-ion battery. BEV, battery electric vehicle. PHEV, plug-in hybrid electric vehicle. ESS, energy storage system.

The lithium anthroposphere life cycle is divided into five stages: mining, refining, manufacturing, use, and waste management, which is shown in Fig. 2. All the flows and stocks throughout the entire lithium anthroposphere life cycle are involved in the system boundary. The spatial boundary is set to be mainland China, which accounts for over 60% of the global lithium chemical production and 70% of the global LIB production [55]. This means that the results of lithium MFA in China could provide valuable insights for improving the material efficiency of the global LIB supply chain. The temporal boundary is from 2015 to 2021, reflecting the latest status of lithium metabolism with sufficient data.

Lithium flows and stocks throughout the anthroposphere life cycle are described as follows. Currently, lithium is mined from two sources: hard rock ores, and brines [56]. Hard rock ore deposits include spodumene, lepidolite, and petalite. Brines are obtained from the salt lake deposits. Lithium can also be extracted from clays, oil filed, and seawater [57]. None of these sources is currently commercially exploited, thus they are not covered in the system boundary. Lithium minerals are converted into several basic chemicals, including lithium carbonate, lithium hydroxide, lithium chloride, and lithium concentrate in the refining stage [27]. The production process of the chemicals varies based upon the deposit types and processing technologies. For example, lithium chloride could be produced with lithium carbonate as feed or directly extracted from brines. There are two kinds of basic chemicals: battery-grade with 99.5% purity and technical-grade with 99% purity. Battery-grade basic chemicals are used to produce the LIB cathode materials and electrolytes, including lithium cobalt oxide (LCO), lithium manganese oxide (LMO), lithium iron phosphate (LFP), lithium nickel cobalt manganese oxide (NCM), lithium nickel cobalt aluminum oxide (NCA), and lithium hexafluorophosphate (LiPF_6_), etc. [10]. Technical-grade basic chemicals can be used to produce several derivatives such as lithium metal or directly applied in the manufacturing stage of non-LIB products.

After being manufactured by cathode materials, electrolytes, and other components, LIB would be installed in several applications, including battery electric vehicles (BEVs), plug-in hybrid electric vehicles (PHEVs), energy storage systems (ESSs), phones, laptops & tablets, and other electronics such as power tanks, cameras, electric tools, robots, drones, etc. In addition to LIB applications, lithium is used in other products, including ceramics & glass, lubricating greases, brazing flux, desiccants, pharmaceuticals, alloys, polymers, etc. These products are accumulated in the use stage to become in-use stocks and then flow into the waste management stage when they reach the ends of their lifespans, becoming the old scraps. In the waste management stage, scraps could be disposed into the environment, reused within the current system or in other systems, or recycled after collection.

### 2.2 Accounting methods of lithium flows and stocks and data sources

For the lithium embodied in the commodities covered in the mining, refining, and manufacturing stages, there are four types of flows: production, import, export, and consumption. Production data of lithium-containing commodities were obtained from relevant statistical databases and reports of minerals [58], basic chemicals [59,60], chemical derivatives [61], [62], [63], [64], [65], LIB products [66], [67], [68], [69], [70], [71], and non-LIB products [27,66,72]. Import and export data were obtained from the China customs data statistics platform based on the customs codes for different commodities, as listed in Table A1 in the Appendix [60,73,74]. Then all the lithium flows in production and international trade are converted based upon the lithium contents of corresponding commodities. The lithium contents of minerals, chemicals, and non-LIB products are calculated by the mass fraction of lithium in the corresponding chemical composition. Lithium contents of the LIB products are obtained by multiplying the LIB capacity of each product with the lithium content of LIB [27]. Lithium contents are listed in Table A1. For each type of commodity, lithium consumption flow could be calculated based on the material balance principle, as Eq. 1:

(1) CO=(PO+IM−EX)*LC

where CO is consumption flow; PO is production amount; IM is import amount; EX is export amount; LC is lithium content.

Here, the consumption of each commodity refers to its apparent consumption, which may not all flow into downstream production. Some commodities could become inventories or losses during the processing stage, i.e., new scrap, without entering the market. Because none of the inventory and loss data is available, these two flows were not considered separately. Following the basic principle of MFA, the input flows are equal to the output flows in the mining, refining, and manufacturing stages. Thus, the net addition to inventory and loss in each stage can be obtained by calculating the difference between the consumption of raw materials and domestic production of downstream commodities. When consumption of raw materials is smaller than downstream production, the net addition to inventory is treated as the input flows assuming that no loss exists. Otherwise, the net addition to inventory and loss are added together as the output flows.

In the use stage, the in-use stock is calculated by using the top-down approach [75], which is the cumulative difference between input flow (final consumption of products) and output flow (scrap), shown in Eq. 2.

(2) $$\;{\text{ST}}_{i}={\text{ST}}_{2014}+\sum_{k=2015}^{i}\left({\text{CO}}_{k}-{\text{SC}}_{k}\right)$$

Where, $ST_{i}\;$is the lithium in-use stock in year *i*; ${\text{CO}}_{k}$ is the lithium consumption in various products in year *k*; $SC_{k}$ is the lithium scrap generated in year *k*.

The scrap flows are modeled by calculating the discard probability of the product being used. We used the Weibull lifetime distribution to estimate the discard probability [76,77]. The parameters of the Weibull lifetime distributions of different commodities were collected from various sources, as listed in Table 1. For commodities not included in Table 1, their maximum lifespans are no longer than one year. Thus, the annual scrap flow of these short-life commodities equals the consumption flow.

(3) $${\text{SC}}_{k}=\sum_{t}^{k}{\text{CO}}_{t}*\frac{\alpha }{\beta }*{\left(\frac{k-t}{\beta }\right)}^{\alpha -1}*e^{-{\left(\frac{k-t}{\beta }\right)}^{\alpha }}$$

where, ${\text{SC}}_{k}$ is the scrap generated in year *k*; ${\text{CO}}_{t}$ is the lithium consumption in various products in year *t*; α is the shape parameter; β is the scale parameter, which is the average product lifespan.

**Table 1:** **The lifespan distributions of various products**.

Species of products	Shape parameter α	Scale parameter β	Refs.
EVs	4.2	10	Sun, Hao [19]
Phones	3.1	2	Nassar [78]
Laptops & tablets	2.6	3	Nassar [78]
Other electronics	2.4	4	Sun, Hao [19]
ESSs	2.1	8	Sun, Hao [19]
Glass & ceramics	4	20	Miatto, Schandl [79]

In the waste management stage, only the recycling and reused flows of LIB products are considered in this study. The scraps of non-LIB products are assumed to be the flow lost into the environment since the recycling potential of these products is quite limited. The recycling data of LIB were collected from relevant institutions [80,81]. Current commercial recycling of LIBs mainly focuses on the recovery of cobalt and nickel because the recovery of lithium is relatively not profitable. Thus, LIB recovery and lithium recovery are not linearly correlated. Recycled lithium amount is obtained from relevant statistical reports [82]. Currently, lithium is all recycled through the hydrometallurgy process, thus the recycling flow direction is from LIB scrap to lithium carbonate production [83,84]. The reused flow of LIB products refers to the echelon use of automobile LIBs. When the capacity of automobile LIB decreased to less than 80% of the initial value, it should be retired from the EVs [85]. The retired automobile LIB could be applied in the stationary ESS based on the capacity left, which is called echelon use [86]. The data about echelon use were obtained from reports of relevant ESS project stakeholders [87].

### 2.3 Uncertainty analysis

**Fig. A1:**
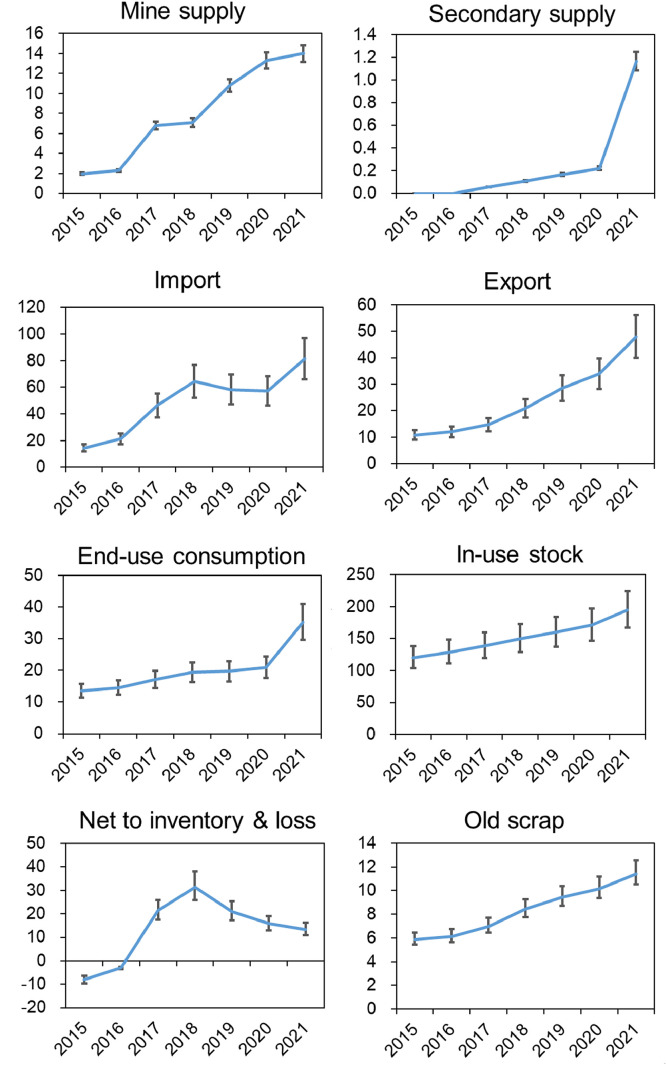
**Uncertainty analysis results of lithium flows and stocks in China**.

The input variables used for accounting the flows and stocks are uncertain due to the statistical errors and subjectivity of assumption. To quantify the impacts of uncertainties of input variables on the MFA results, we performed the Monte Carlo simulation 10,000 times. The uncertainties of input variables were modeled by the normal distribution [88]. Referring to the previous literature, we assumed that the uncertainties of statistics data (production, international trade, recycled material, etc.) are lower than those of model assumptions (lithium contents, product lifespans, etc.). Because of lacking sufficient data for real uncertain ranges, the standard deviation of statistics data and model assumptions were assumed to be 2% and 5%, respectively [89]. Results of uncertainty analysis are shown in Fig. A1 in the Appendix.

## 3 Results

**Fig. 3:**
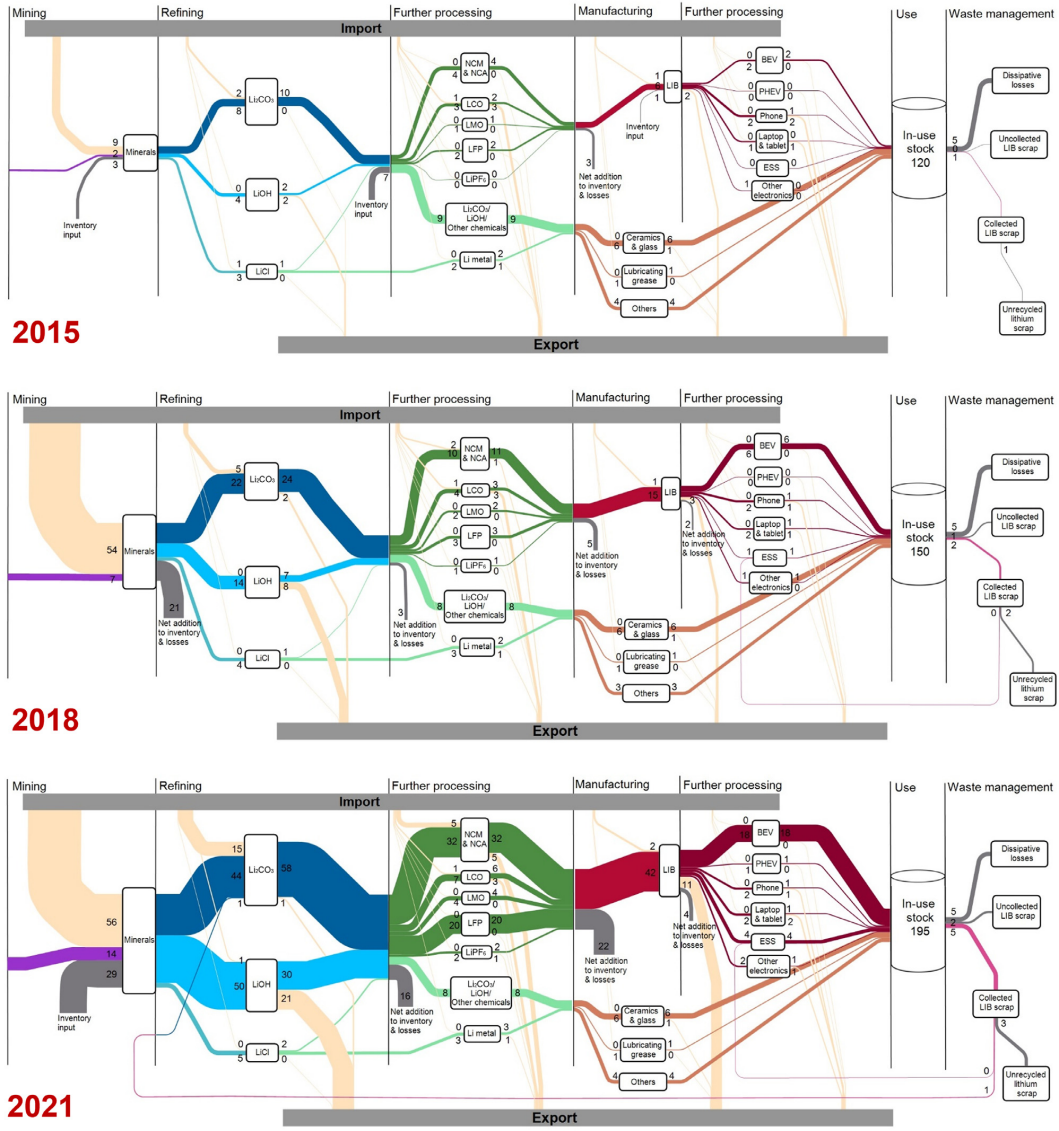
**Lithium flows and stocks in China for the years 2015, 2018, and 2021**. The flow direction is from left to right. The width of each flow is proportional to its corresponding value. All the values are rounded to one significant digit in kiloton lithium metallic equivalent unit. Vertical black lines indicate processing stages. Rectangles indicate lithium-containing commodities.

**Fig. 4:**
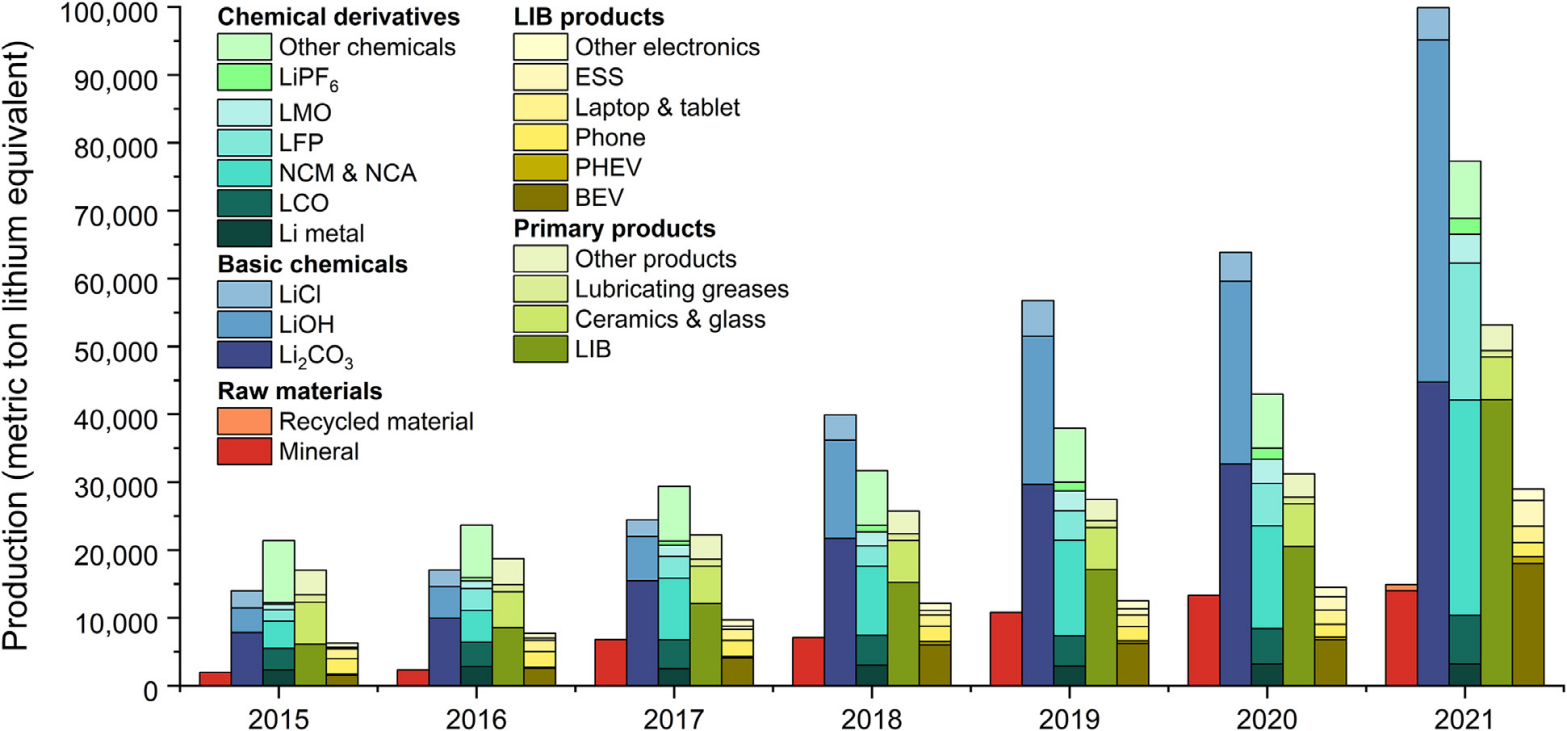
**Production structure of lithium-containing commodities in China**. The five columns of each set of bar charts show the supply structures of five categories of commodities: (1) raw materials, (2) basic chemicals, (3) chemical derivatives, (4) primary products, and (5) LIB products.

**Fig. 5:**
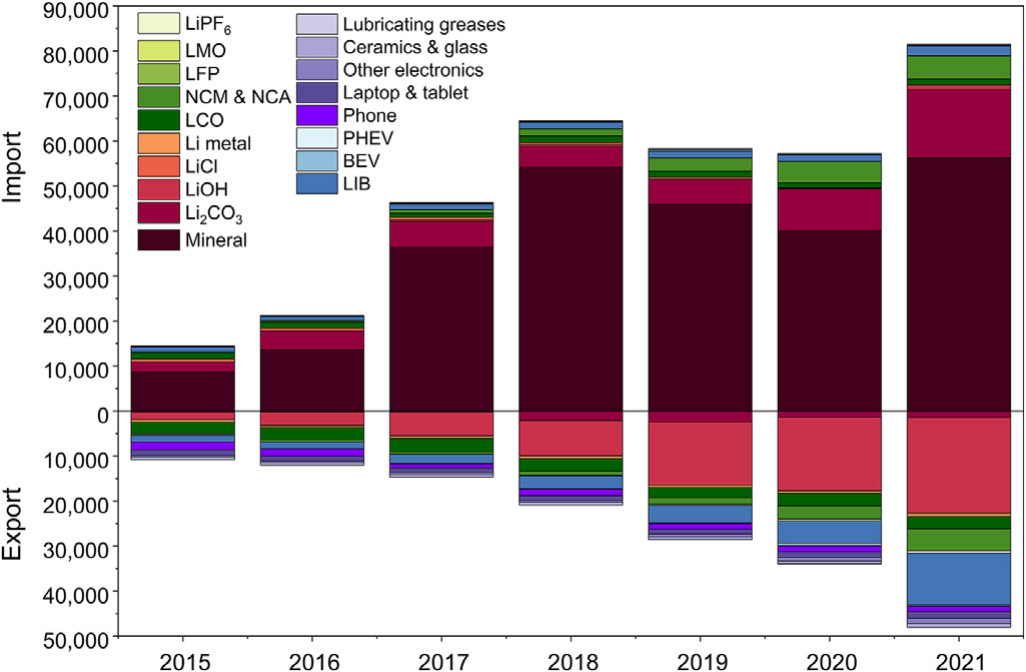
**Import and export of lithium-containing commodities in China**. The bars above and below the zero-value line show the import volume and the export volume, respectively. Unit in metric ton lithium metallic equivalent.

**Fig. 6:**
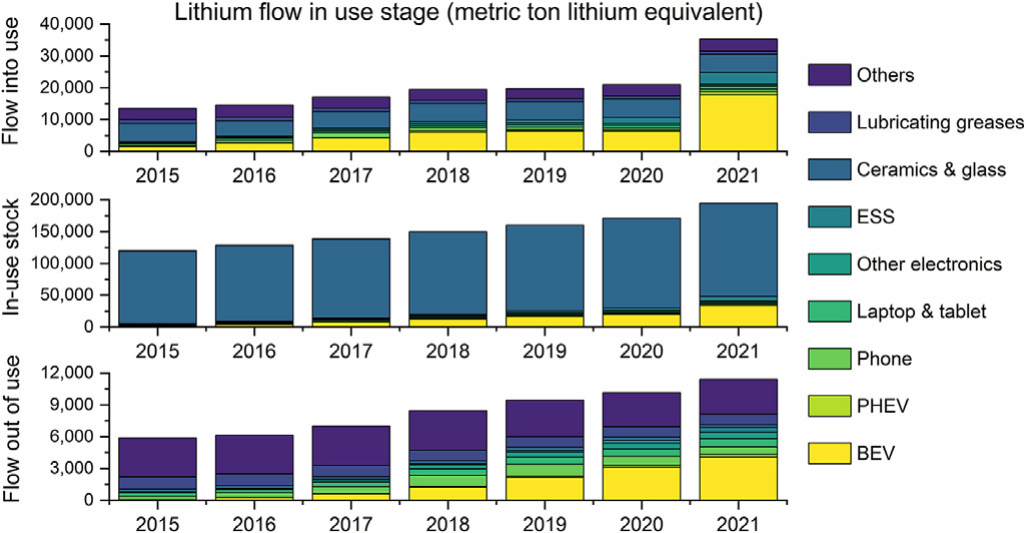
**China's lithium flow in the use stage**. Top-to-bottom three lines of subfigures indicate annual lithium flow into use, in-use stock, and flow out of use, respectively.

The results of tracing the material flow and stock of lithium in China from 2015 to 2022 are presented in this section, which is divided into the following four sections. Section 3.1 presents the overview of the lithium anthroposphere cycle in China, where major results are shown in Fig. 3. Fig. 4, Fig. 5 show the production-side and international trade-side of lithium flow in various commodities, respectively, which are presented in Sections 3.2 and 3.3. Lithium flows in the use stage, including the end-use consumption, in-use stock, and waste, are presented in Section 3.4, which are shown in Fig. 6. All the values without references appearing in the following are the results of our own calculations. Unless otherwise specified, all the units of the amounts below are lithium metal equivalents.

### 3.1 Overview of lithium flows from minerals to end-use products in China

As Fig. 3 shows, from 2015 to 2021, the lithium flow has increased significantly in China due to the rapid development of the EV industry. The total flow into the refining stage increased seven-fold to 100 kt in 2021 compared to 2015. This increase was driven by the development of the LIB product market, mainly the EV market. Correspondingly, the domestic and international trade flows of lithium carbonate, lithium hydroxide, LIB cathode materials, and electrolytes all have significant growth. Contrary to this, lithium flows into non-battery products, e.g., ceramics and glass, and relevant raw material markets were nearly unchanged.

On the other side, the structure of China's lithium flow has not changed much. China has a comprehensive domestic lithium supply chain with the production capacity of all types of lithium-containing commodities. There is a significant mismatch between China's domestic mineral supply and the production capacity of downstream commodities. China is the world's fourth largest lithium reserve owner, whose reserve accounts for 7% of world reserves. 80% of China's lithium reserves are located in brines in Qinghai and Tibet provinces. The harsh local geography and low mineral grades (high magnesium-to-lithium ratio) make it difficult and expensive to mine. In 2020, 60% of China's domestic lithium supply came from lithium spodumene and lithium lepidolite mines [60]. Although domestic supply has also increased, China's import dependency on minerals (ratio of imports to the sum of imports and domestic production) remained at a high level during 2015–2021, ranging from 75% (in 2020) to 88% (in 2018). Because other countries do not have such large refining and manufacturing capacity, China has always been the world's only importer of lithium minerals, vast majority of which were lithium spodumenes from Australia.

Such large-scale import was to serve not only the domestic soaring demand for LIB products but also for export demand. Cumulatively, 55% of the lithium went to the export market while the rest went to China's domestic consumption side. There was no significant change in the structure of supply and demand for various commodities. Commodities with the largest shares of cumulative export demand in the total demand were laptops & tablets (64%), phones (62%), other electronics (54%), lithium hydroxide (53%), and LCO (46%) during the study period. The manufacture of these commodities has always been export-oriented. Such shares for other commodities are all smaller than 25%, indicating that their production was driven by domestic demand. China's domestic lithium consumption flow into the use stage increased from 14 kt to 35 kt, making the in-use stock reach 195 kt in 2021.

### 3.2 Production structure of five categories of lithium-containing commodities

As Fig. 4 shows, the production of all categories of commodities maintained a steady growth from 2015 to 2020, and then had a sharp growth in 2021 due to the unexpected growth of the global EV market this year. China's domestic production of lithium raw materials reached 15 kt in 2021 with an annual growth rate of 40% in the past seven years. From 2015 to 2020, China's domestic production of lithium raw materials were all from mineral deposits. In 2021, 94% of raw materials were minerals, while 6% were recycled materials.

Lithium flow in the production of basic chemicals reached 100 kt in 2021 with an annual growth rate of 39%. In terms of the commodity types, the production of lithium hydroxide experienced the fastest growth, with an annual growth rate of 55%. Lithium carbonate had been always the largest share of basic chemical production before 2020, but being surpassed by lithium hydroxide production in 2021. The share of lithium hydroxide production in the total basic chemical production jumped to 50% in 2021 from the last year and that of lithium carbonate reduced from 52% to 45%. The rapid growth of lithium hydroxide production is mainly due to the popularity of nickel-rich LIB cathode materials, which has the highest energy density and could be only produced by lithium hydroxide.

The production of lithium chemical derivatives increased from 21 kt in 2015 to 77 kt in 2021, with an annual growth rate of 24%. The cumulative production of NCM & NCA during the study period was the largest, accounting for 34% of the total production of chemical derivatives. From 2015 to 2020, the production of NCM & NCA had experienced the fastest growth, of which the annual growth rate was 44%. The growth rate of LFP production was the second largest, with a figure of 24%. From 2020 to 2021, LFP production increased by 230% to 20 kt, far outpacing the growth rate of NCM & NCA (110%).

Lithium applied in the production of the primary products increased from 17 kt to 53 kt, which all came from the incremental production of LIBs. The annual growth rate of LIB production was 38% and that of other primary products was −1% from 2015 to 2021. The production of LIB products increased from 6 kt in 2015 to 29 kt in 2021. In 2015, the top 3 LIB products with the largest production shares were: phones (36%), BEVs (25%), and laptops & tablets (23%). In 2021, the share of BEV production in the total LIB product production increased to 62%. ESS accounted for the second largest share, which was 13% and those of phones and laptops & tablets reduced to 7% and 8%, respectively.

### 3.3 International trade structure of lithium-containing commodities

Lithium flow in importing commodities experienced volatile growth, increasing from 14 kt in 2015 to 64 kt in 2018, then decreased to 57 kt in 2020, and jumped again to 79 kt in 2021 (Fig. 5). This trend was dominated by the changes of the imported lithium minerals which kept being the largest import flow (56 kt in 2021). The amounts of imported lithium carbonate and NCM & NCA were the second and third largest in 2021 with figures of 15 kt and 5 kt, respectively. The imported amount of NCM & NCA experienced the fastest growth, with an annual growth rate of 120% during the study period.

China's lithium export flow increased stably, from 11 kt in 2015 to 48 kt in 2021, but less than the import flow. Exports of LCO, phones, and lithium hydroxide accounted for the largest shares in the total export flow in 2015, which were 23%, 15%, and 14%, respectively. In 2021, the commodities with the top 3 largest shares in export flow became lithium hydroxide (44%), LIBs (24%), and NCM & NCA (10%).

### 3.4 Lithium flow in the use stage

China's lithium flow into the use stage grew steadily and reached 21 kt in 2020 with an annual growth rate of 9%, and then increased by 69% to 35 kt in 2021 (Fig. 6). The sudden spike in lithium final consumption flow in 2021 was mainly driven by the EV market. In 2021, lithium flow into the BEV use and PHEV use was 18 kt and 1 kt, with a year-on-year increase of 180% and 140%, respectively. As a comparison, the lithium flow in these two uses was only 1.6 kt and 0.1 kt in 2015. ESS consumption also increased rapidly, from 0.2 kt in 2015 to 3.5 kt in 2021. The consumption of other LIB products grew relatively slowly from 1 kt in 2015 to 2 kt in 2021. As such, the consumption of non-LIB products remained nearly constant with a figure of 10 kt.

Lithium in-use stock in China was 195 kt in 2021 with an annual growth rate of 8%, reaching 13% of China's natural lithium reserve [58]. In 2015, most of the lithium in-use stock was from ceramics & glass, BEVs, and laptops & tablets in China, which accounted for 95%, 2%, and 1% of the total (120 kt). Ceramics & glass remained to be the largest share of lithium in-use stock due to its long lifespan, while its share reduced to 75% in 2021. Lithium in-use stock in BEVs and ESSs experienced the fastest growth, accounting for 17% and 3% of the total in-use stock in 2021 as the second and third largest applications. Scrap flow out of the use stage was 11 kt in 2021, nearly double that in 2015 (6 kt). Scrap flow from non-LIB products had been the largest until 2019, and its share in the total scrap flow reduced from 87% in 2015 to 51% in 2021. Since 2020, LIB products became the largest source of lithium scrap flow due to the rapid growth of BEV use, which accounted for 35% of the total scrap generated in 2021.

## 4 Discussion

### 4.1 Optimizing inventory and processing, and developing substitutes

**Fig. 7:**
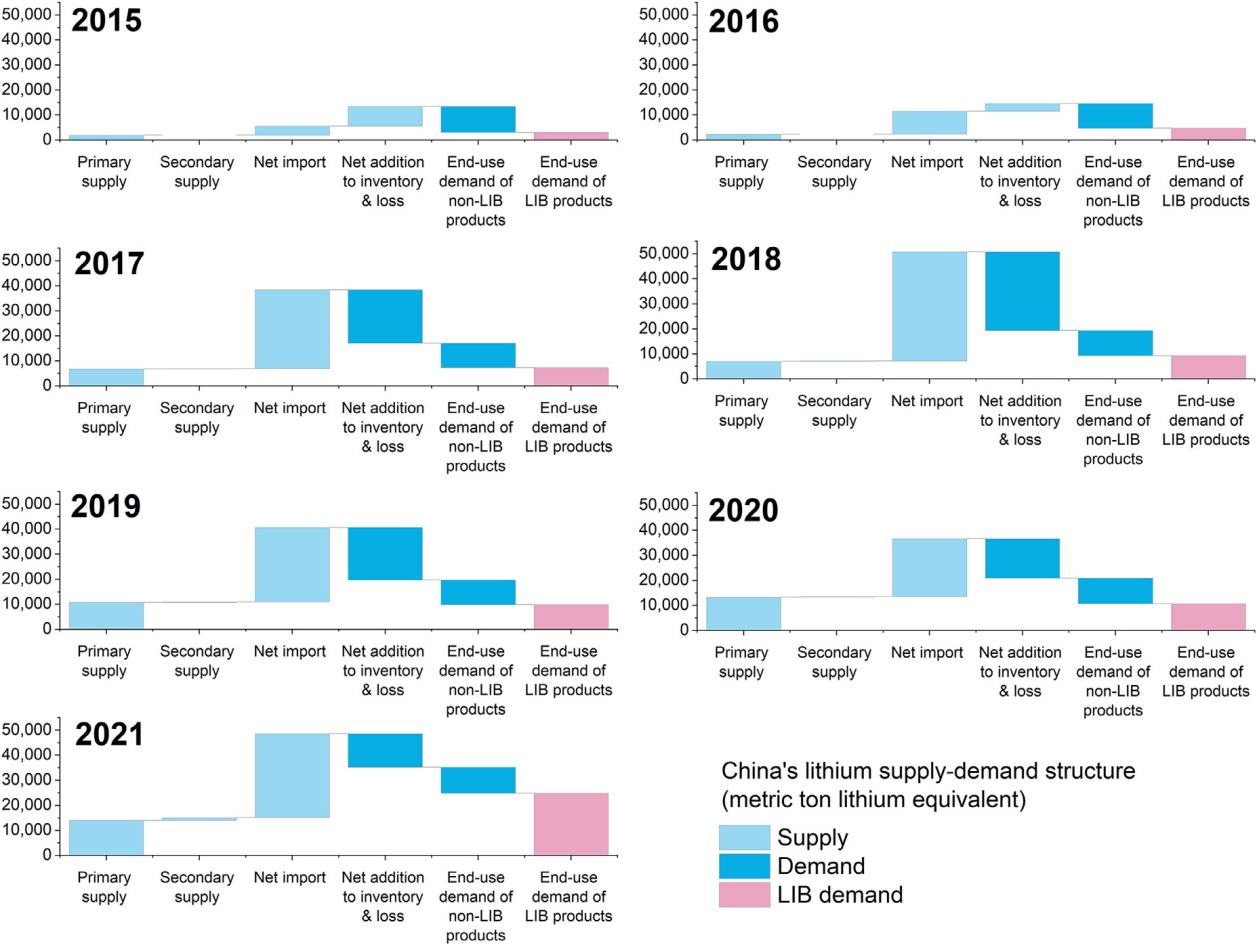
**Bridge chart of China's lithium supply-demand balance from 2015 to 2021**.

Based on the collation and refinement of the lithium material flow map, China's lithium supply-demand balance was constructed, as shown in Fig. 7. On the supply side, net imports kept being the largest source except for the year 2015. From 2015 to 2021, net import dependency, which is defined as the share of net import on the total supply, ranged from 27% to 86% (Fig. 10). The average net import dependency was 66% from 2015 to 2021, reflecting a great threat to the security of the national resource supply. On the demand side, a significant portion of lithium supply has not been effectively used to serve the demand of the end-use market but became inventories or was wasted in the processing stages. In 2015 and 2016, due to the boom of the EV market, the lithium supply-demand balance was in a tight state, resulting in that there were more inventories as supply than demand. Since 2017, China's lithium market kept in a state of oversupply along with the steady growth of the EV market. The share of lithium flowing into inventories and losses in the total demand peaked at 62% in 2018, and then gradually dropped to 28% in 2021. From 2015 to 2021, the cumulative lithium flow into inventories and losses reached 103 kgtons. As a comparison, China's cumulative mining production was only 56 kt. The great amount of lithium flow into inventories and losses in the processing stages indicates the huge potential for optimizing China's lithium supply chain.

**Fig. 8:**
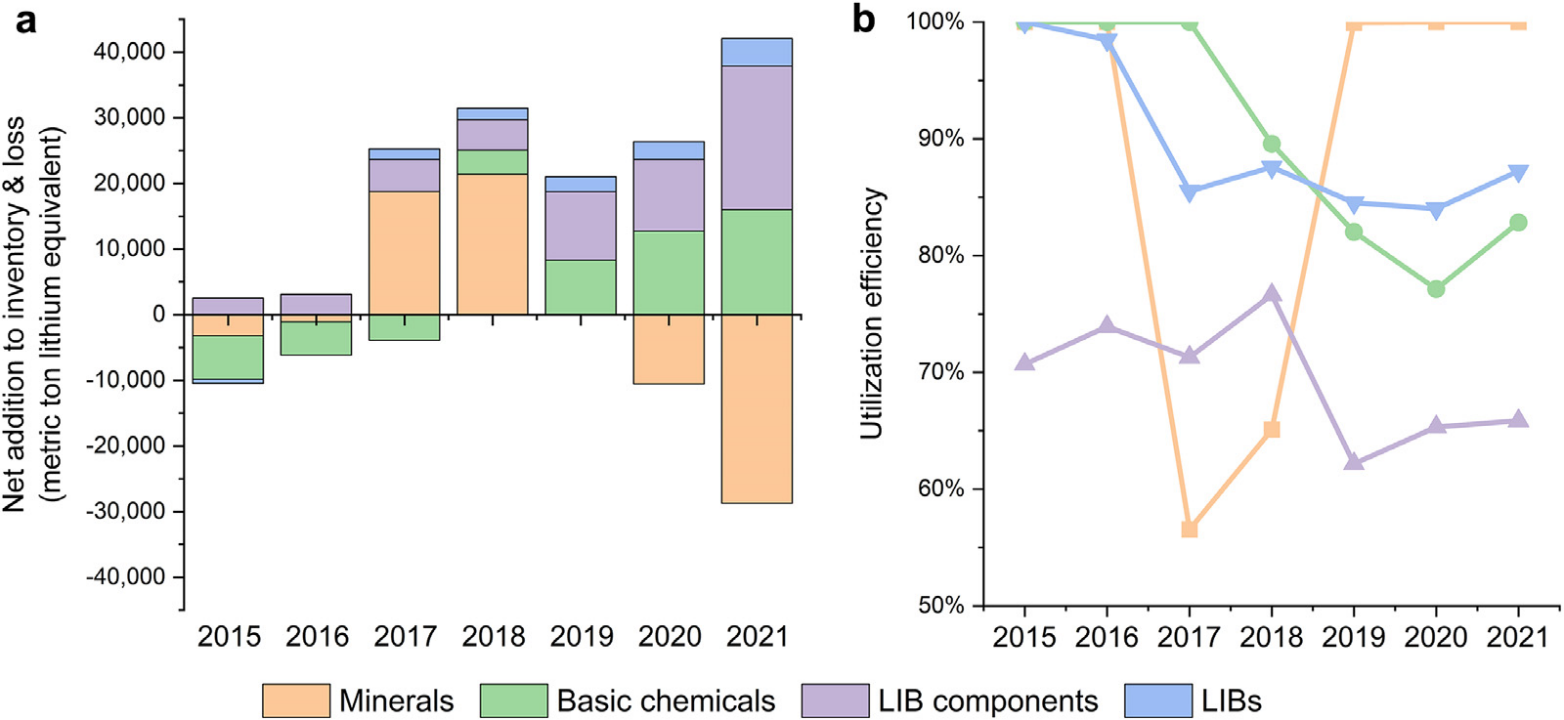
**(a) Net addition to inventory and loss and (b) utilization efficiency of four categories of lithium-containing commodities**. (a) Positive values indicate that the commodities produced in that year do not enter the market for further processing but were converted into inventories or were lost in processing. A negative value indicates that the inventories of commodities that were produced in the past years enter the market for further processing. (b) Utilization efficiency is defined as the ratio of lithium flow in commodities entering further processing stages to lithium flow in the apparent consumption of corresponding commodities.

In terms of commodity types, most of the lithium flow into inventories and losses were LIB components (including cathode materials and electrolytes) and basic chemicals (including lithium carbonate, lithium hydroxide, and lithium chloride), which accounted for 63% and 27% of the cumulative flow into inventories and losses during the study period, respectively (Fig. 8a). Considering the different market sizes of different commodities, we assess the utilization efficiency of various commodities to make comparisons. Here the utilization efficiency is defined as the ratio of lithium flow in commodities entering further processing stages to lithium flow in the apparent consumption of corresponding commodities. These results are shown in Fig. 8B. Utilization efficiency of minerals used to be the lowest in 2017 and 2018, which were 57% and 65%, respectively. In other years, the supply and demand of minerals were in a tight balance. The utilization efficiency of basic chemicals and LIBs have been maintained in a relatively stable state, which in most cases was above 80%. While for LIB components, their utilization efficiency has always been in a bad state, ranging from 60% to 80%. It indicates that there is a serious longstanding overcapacity and weak processing technology in the LIB component industry in China. The underlying causes of the overcapacity issue could be vicious production competition among the LIB component suppliers, the presumptuous pursuit of the speed of capacity expansion, and the lack of industry entry thresholds. Addressing these issues requires an in-depth analysis of the relevant influencing factors and considerable policies for governance.

**Fig. 9:**
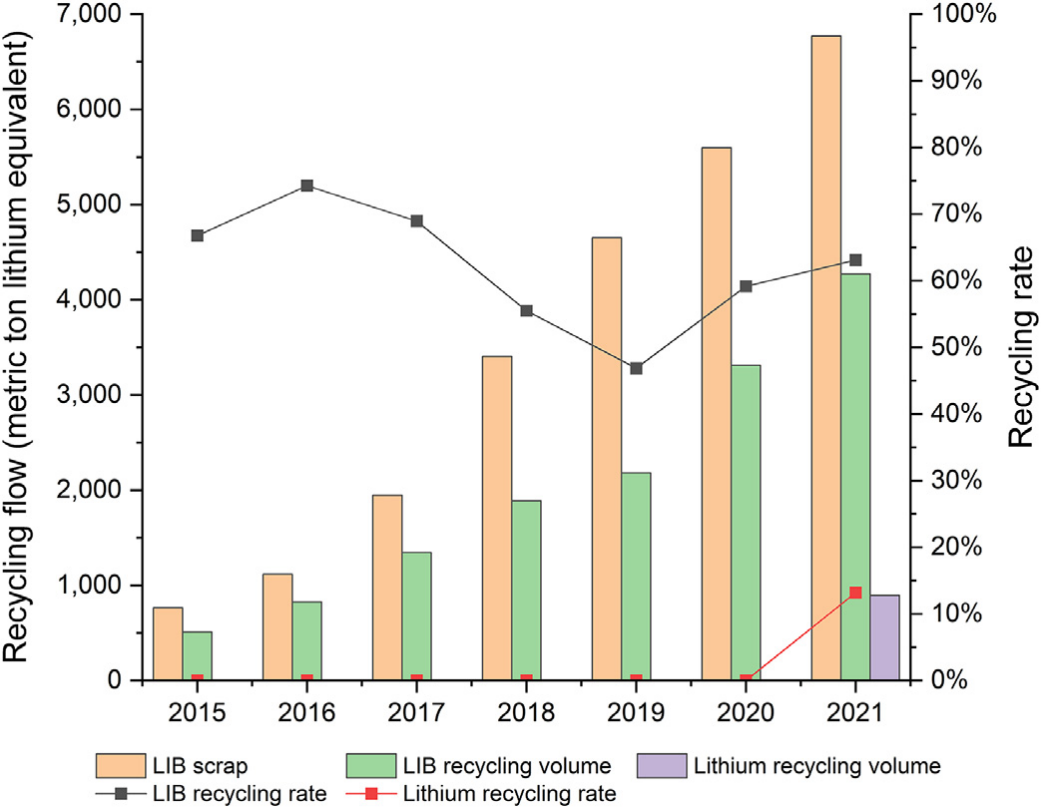
**Lithium recycling flow in China from 2015 to 2021**. Bars show lithium flow in LIB scrap, recycled LIB, and recycled lithium from LIBs. The corresponding vertical coordinate is on the left. Lines show the end-of-life recycling rate of LIBs and lithium. The corresponding vertical coordinate is on the right.

**Fig. 10:**
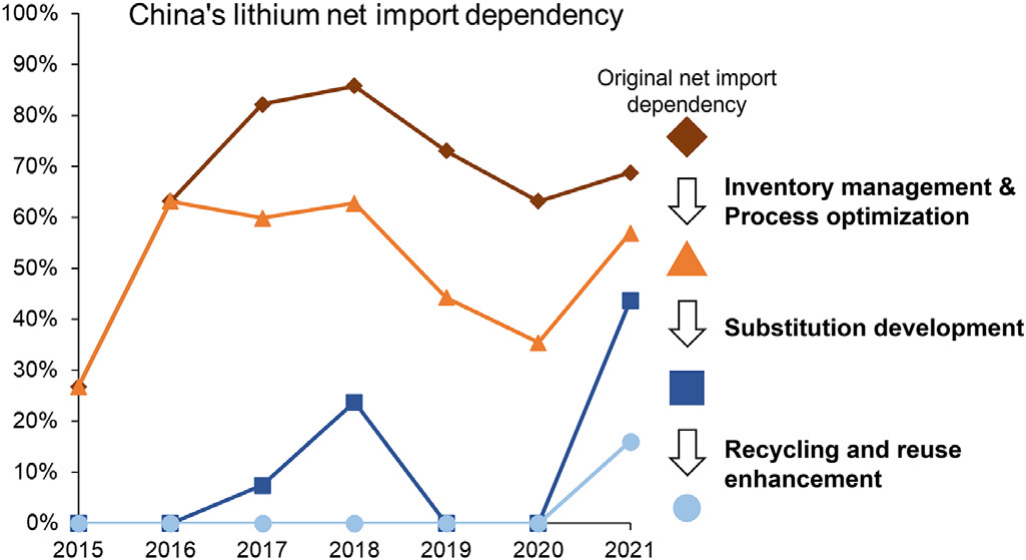
**Potential effect of three strategies on China's lithium net import dependency**. Four lines indicate the annual China's lithium net import dependency under the overlapping effect of various strategies.

Improving material utilization efficiency could help reduce the consumption of lithium resources and avoid market fluctuations due to the demand distortion caused by high inventories. More importantly, it can contribute to reducing the import risk by avoiding the irrational demand. If utilization efficiency of all the commodities could reach 100% by improving processing and optimizing inventory management, the net import dependency of China's lithium supply could be reduced to the range of 27% to 63%, assuming other supply sources are unchanged (Fig. 10).

Lithium can be substituted in the non-LIB products by other elements, such as calcium, aluminum, sodium, etc. But it is hard to be substituted in batteries without affecting product performance [58]. If such substitution is vigorously developed along with the improved commodity utilization efficiency, the net import dependency of China's lithium supply could be at the range of 0% to 44% to meet domestic consumer demand for LIB products (Fig. 10). Along with the popularity of clean energy products, such as EVs, lithium will become increasingly critical in the national economic system. Consequently, more research efforts should be made to develop innovative technologies so that lithium can be substituted by non-critical metals in non-LIB applications.

### 4.2 Enhancing lithium recycling and reuse system

Recycling is an essential way for improving supply chain resilience, resource conservation, and reducing the negative environmental impacts of material processing [90], [91], [92]. In recent years, LIB recycling has gained global attentions from social, commercial, and academic communities [43,93]. The continuous investment in R&D of LIB recycling technologies, as well as the gradual improvement of regulations, made great progress in the LIB recycling industry, especially in China. Because China's LIB recycling costs are among the lowest in the world [94], the scale of commercial LIB recycling in China has been developing by leaps and bounds, with an annual growth rate of 43% from 2015 to 2021. In 2021, 4 kt (lithium content) of LIB was collected and recycled, accounting for 63% of the total LIB scarp flow.

From 2016 to 2019, the LIB recycling rate kept declining, which showed the opposite trend to the change of LIB recycling volume. Before 2019, most of recycling operators focused on the recovery of consumer electronics batteries because the amount of end-of-life power batteries was still relatively limited and the corresponding recycling technology and regulations were not yet perfect. The growth of LIB recycling volume was in line with the growth of consumer electronics battery scrap but was smaller than that of total scrapped LIBs, which was mainly driven by power LIB scrap. From 2019 onwards, the continued good performance of the power LIB market has ignited the enthusiasm of the investors. Coupled with the influence of accelerating policies and regulations that tend to mature, the growth of power LIB recycling capacity entered a blowout phase. The growth of LIB recycling volume began to be higher than the growth of LIB scrap, making the LIB recycling rate rebound.

Nevertheless, our results indicate that even with a certain scale of LIB recycled annually, the recovery of lithium was in its early stage. The lithium recovery rate is much lower than the LIB recovery rate because recycling firms cannot gain profits from recycling lithium. The unit price ($/kg) and content (kg/kWh) of lithium in LIBs are much lower than other valuable metals such as cobalt and nickel [43,95]. In addition, the process of recovering lithium is more complicated than recovering other valuable metals, which can only be achieved by hydrometallurgy technology but not pyrometallurgy technology, further increasing the cost [96]. These reasons led to a lithium recycling rate of approximately zero before 2020. Since the second half year of 2021, hiking lithium price stimulated commercial lithium recycling activities, leading to annual lithium recovery of 0.9 kt. The recycling rate of lithium from end-of-life LIB scrap reached 13% in 2021.

It should be noticed that the growth of lithium recovery is induced by a 10-fold surge in lithium prices (Fig. 1), which is not sustainable. Lithium price is expected to decrease to the pre-surge level since the future lithium raw material production capacity will gradually grow. Therefore, if the lithium recycling industry wants to operate in the long term, these companies need to invest more in recycling technologies so that the cost of lithium recycling can be reduced. In this regard, the Chinese government may provide financial subsidies to incubate such lithium recycling technologies so that more investors would engage in this recycling efforts.

Another secondary supply source—echelon use, has not yet played a significant role in supporting lithium supply. Recorded echelon use of LIBs in China started in 2017, with a figure of 0.05 kt. This figure reached 0.3 kt in 2021, accounting for 7% of the total ESS production. Currently, construction of echelon use projects is mainly driven by policy. Similar with the recycling system, the cost of testing and reassembly of retired automobile LIBs is higher than the purchase cost of energy storage LIBs, leading to that these firms cannot obtain commercial profits. In addition, concerns about the safety of reusing end-of-life LIBs further hinder the marketability of echelon use. Therefore, more efforts should be initiated so that more advanced battery technologies can be developed with more regulatory support.

As Fig. 10 shows, together with inventory management, process optimization, and substitution development, if all the end-of-life LIBs could be effectively recycled or reused, China's lithium net import dependency could reduce to 0% during the study period, achieving complete self-sufficiency of lithium supply. In 2021, the net import dependency could reduce to 16% to meet soaring LIB demand. Enhancing the use of domestic resources has been a major strategy for reducing China's import risk [97,98]. However, due to the long lead time required for exploration and development of mineral projects, strategies to increase domestic supply will not be effective in the short term and will face uncertainty on the mining cost. Our results indicate that improving material utilization efficiency deserves more attention from all the stakeholders.

## 5 Conclusion

As the core energy storage equipment in electrified mobility and power system, LIB is one of the most critical technologies for the low-carbon energy transition. Correspondingly, the sustainability of LIB supply chain received a broad concern because it heavily relies on many critical materials, e.g., lithium, and cobalt, as the input raw materials. However, the up-to-date status of material utilization efficiency and supply security has not been well understood. To fill this gap, this study employed the MFA method to comprehensively investigate lithium metabolism along its LIB supply chain in China from 2015 to 2021. Results indicate that the lithium flow scale has been growing rapidly in China due to the skyrocketing development of the EV industry. Domestic production of basic chemicals reached 100 kt in 2021 with an annual growth rate of 39%. End-use consumption increased by 160% to 35 kt from 2015 to 2021, making in-use stock in China reach 195 kt. As imports and exports both grew steadily, China kept being a net importer of lithium, leading to more concerns about stable resource supply.

A significant finding of this study is that a cumulative 92 kt of lithium was converted into inventories or was lost during the processing stage from 2015 to 2021. This is a serious problem that has been neglected in previous studies and reports for a long time. If this part of the waste is reduced to zero, China's net import dependency can be reduced from 27%−86% to 27%−63% to meet domestic end-use demand for lithium. In addition, considering the possible substitutions to lithium in non-LIB applications, China's net import dependence could even be reduced to 0%−44% to meet end-use demand for LIB products.

Another key finding is that even recycling system for LIBs has been continuously improved, the recycling rate of lithium from LIB scrap is still low, with a figure of only 13% in 2021, much less than that of LIBs (63%). There is a high mismatch between the recycling of lithium batteries and the recycling of lithium because the recycling benefit of lithium is much lower than that of other valuable materials in LIBs. Enhancing the recycling and reuse system could help China's net import dependence further reduce to 0%−16%. These findings confirm that improving utilization efficiency can effectively help strengthen national lithium resource security and should be given a high priority.

Statistical errors in the underlying data and academic assumptions may influence the quantitative results of this study since they may not be consistent with the real material flows. But we have full confidence in our conclusions, which are verified by the uncertainty analysis. The key findings in this study provide valuable policy insights to make appropriate lithium resource management policies, such as optimizing inventory management, improving processing efficiency, and enhancing lithium recovery systems, so that a resilient and sustainable LIB supply chain can be established. The specific implementation strategies and potential impacts of each measure call for further studies.

## CRediT authorship contribution statement

**Xin Sun:** Conceptualization, Methodology, Formal analysis, Investigation, Data curation, Writing – original draft, Writing – review & editing, Visualization. **Han Hao:** Conceptualization, Methodology, Supervision, Writing – review & editing, Funding acquisition. **Yong Geng:** Methodology, Supervision, Writing – review & editing, Funding acquisition. **Zongwei Liu:** Investigation. **Fuquan Zhao:** Writing – review & editing, Funding acquisition.

## Declaration of competing interest

The authors declare that they have no conflicts of interest in this work.

## Figures and Tables

**Table A1:** **The HS codes and lithium contents of lithium-containing commodities**.

Commodity	HS code	Lithium content	Unit
Brines	/	0.12	wt%
Ores	25,309,099	0.56	wt%
Lithium carbonate	28,369,100	19	wt%
Lithium hydroxide	28,252,010	29	wt%
Lithium chloride	28,273,910	16	wt%
Lithium concentrate	25,309,099	2.9	wt%
LMO	28,416,910	3.8	wt%
LCO	28,419,000	7.1	wt%
LFP	28,429,040	4.4	wt%
NCM	28,429,030	7.2	wt%
NCA	28,429,060	7.2	wt%
LiPF_6_	28,269,020	4.5	wt%
Glass and ceramics	69 & 70	1.4E-06	wt%
Lubricating greases	27,101,992	0.33	wt%
LIBs	85,076,000	0.13	kg/kWh
BEV	870,380	6.1	kg/item
PHEV	870,360 & 870,370	1.7	kg/item
Electric bicycles	87,116,000	54	g/item
Phones	85,171,210	1.3	g/item
Laptops	84,713,010	6.9	g/item
Tablets	84,713,010	3.0	g/item
Power tanks	85,076,000	4.7	g/item
Electric tools	950,310	1.3	g/item
Cameras	852,580	0.79	g/item
Wearable electronics	85,183,000	0.07	g/item
Drones	88,022,000	18	g/item
Energy storage systems	/	0.12	kg/kWh

## Appendix

HS code is from the China Customs database [73]. Lithium content is from the collection of previous literature and authors’ own calculations [27,38]. NCM, lithium nickel cobalt manganese oxide. NCA, lithium nickel cobalt aluminum oxide. LCO, lithium cobalt oxide. LMO, lithium manganese oxide. LFP, lithium iron phosphate. LiPF6, lithium hexafluorophosphate. LIB, lithium-ion battery. BEV, battery electric vehicle. PHEV, plug-in hybrid electric vehicle.

The total flow and stock amounts of all types of lithium-containing commodities are selected as the model output to observe the impacts of uncertainties of input variables. Values in figure are in kt lithium metallic equivalent unit. Blue lines represent original results. Error bars represent the uncertainty ranges. Results indicate that impacts of uncertainties of statistics data and model assumptions are acceptable, which would not significantly affect the conclusions of this study. The uncertainties of imports, exports, end-use consumption flows are relatively higher as they are involved with more statistics data and technical parameters for calculating the lithium content. The underlying data and assumptions used to calculate these flows need to be calibrated with focus in subsequent studies.
